# The National Association of Dental Faculties

**Published:** 1897-11

**Authors:** 


ARTICLE VII.

### THE NATIONAL ASSOCIATION OF DENTAL FACULTIES.

EDITORIAL IN *Dental Practitioner and Advertiser.*

Occasionally, unless the proper officers are on deck, the bark that is borne by the strongest current gets into the eddy, and is forced backward with a velocity that carries consternation to the hearts of the stoutest sailors. That was the case with the National Faculties Association this year. Last year, at Saratoga, those who are really desirous of seeing our educational standard advanced were very much encouraged. A decided step forward was taken, and the college skies were brightly illuminated with the rainbow of hope. This year as much ground was lost as had been gained in several, and the outlook is but a sorry one.

For some time it has been observed with feelings of apprehension, that there was a disposition on the part of the representatives of a majority of the colleges to admit everything to membership that made application. The most serious charges were glazed over, and there has not been a serious controversy raised, unless it was over the case of some institution which, either by the state law under which it acted, or by the known character of its promotors, must earnestly favor a high standard. The fact thac there were one, or two, or three colleges already in existence in a city of moderate size, made no difference. Another applicant was promptly admitted, and the weaker it was ethically the more anxious seemed some representatives to have it get in. We do not mean to intimate that any unworthy motive prompted this, for we do not believe that the threatened danger was foreseen. But the consequences are now very forcibly felt, for tbere is a clear majority in the Association opposed to any raising of the requirements, or lengthening of the term.

Perhaps this statement is not quite correct. This year the bad results were due as much either to the treachery, the singular lack of comprehension, or the stupid fatuity of representatives from whom better things should have been anticipated, as to the deliberate efforts of the low-standard colleges. But the result was the same, no matter what the ruling motive. The attempt to sustain a higher preliminary qualification was decisively defeated. The whole matter was stupidly managed on the part of those interested in advancing the standard, in that they allowed the decisive vote to be taken on a minor question of sustaining the chair in an absurd decision, instead of bringing the matter squarely to an issue, and putting every man on record.

Last year, at Saratoga, a definite system of preliminary credits was adopted, which might give some absolute standard. It was the requiring of a certain number of "points" as the qualification for matriculation. This was in reality very simple, although some claimed that they could not comprehend it. With the exception of the common branches of reading, writing, spelling, elementary arithmetic and grammar, every study pursued for one term in any school above the grade of the common elementary or district school, gave a credit of one point. Thus if a student attended any recognized academy or school in which the higher branches were taught, for two terms, and successfully pursued five studies in each term, he was entitled to ten points. The record was to be determined upon the certification of the proper officers of the school. The diligent pupil might accomplish as much in one year as the sluggard would in two, and hence a premium was offered to assiduity. The standard adopted was that which would be about equivalent to one year spent in any good school.

But some of the colleges in the South and West insisted that this was too high. Their people were not sufficiently educated for even this low standard, and they could not comply with these requirements. This was an extraordinary admission to make, but it was gravely urged in open session. It was vain to remind them that there was no excuse for the existence of colleges in regions where there were no students fit for matriculation. They proposed to make dentists anyway, and they must use the only material they could secure.

At Saratoga, when the proposed system was tentatively adopted, the attention of men interested in general education was attracted to the movement. An expert in educational affairs was invited to address the Association upon the subject to which he had devoted his life. In formulating the plan for publication, the Secretary of the association was by vote authorized to consult him as to the list of studies that were to be considered academic. There was no charge that this was ever done, or that there had ever been interference of any kind, but in seeking some subterfuge for the undoing of the work of last year, the point was made that consultation with an expert in educational affairs who was not a member of the Association, although expressly sanctioned in advance by vote of the members, vitiated the whole proceeding, and made the legislation of last year null and void. The chair sustained the point, and ruled that the whole action was unconstitutional, and the laws adopted therefore inoperative and nugatory. An appeal was taken from the decision of the chair,

*and it was sustained.* It was in vain that some of the representatives attempted to show the utter absurdity of the whole thing, and demanded a statement of wherein the action was illegal or unconstitutional. It was not even contended that the consultation of an outsider, if it had taken place, had in any way affected the matter or changed the status. No reason was given why this made the rules adopted unconstitutional. It was simply charged that it was irregular, and the chair so ruled, and he was sustained by vote of the Association. In fact, the chairman said he did not know just where the unconstitutionality lay, but he wanted it that way, and he ruled that way, and it seemed to meet the wishes of the majority, which was undeniable.

The editor of *The Western Dental Journal* in describing the scene in an editorial communication, says:

"At Old Point Comfort it was soon seen that the objection to the advanced requirements in the Faculties Association were in a majority, and that they had the presiding officer with them. A motion was made to reconsider the vote at Saratoga, and upon the point of order that it was illegal, the chairman ruled that it was in order because the former action was unconstitutional; being pressed to give a reason why it was unconstitutional, he could not do so, until a wag on the opposition side suggested that he might declare it unconstitutional because Mr. Melvil Dewey was given the courtesy of the floor to make a statement when the question was pending at Saratoga in 1896, when—Presto!! That fragment of a reason to hang their tattered hopes upon was just what they were looking for, and so this plaint chairman was sustained by a vote of 17 to 7 in declaring that the rules passed at Saratoga were void because—forsooth—an intelligent outsider was asked an opinion!!

The whole proceeding was certainly comical. Usually grave and reasonable dental teachers voted to sustain the laughable decision of the chairman, as if they believed in its regularity, and generally level heads seemed to waver under the determined cries of 'Down with the Examiners,' 'Let them attend to their own affairs.'"

And so the National Association of Dental Faculties is practically without any standard of qualification for entrance into the colleges which compose its membership. It falls back upon the rule of some years ago. As near as can be determined that is as follows:

"The minimum preliminary educational requirement of the colleges of this Association shall be a certificate of entrance to the first year of a High School, or in states that have no High School, of graduation from a grammar school, or its equivalent, to be determined by an examination."

The whole essential part lies in the last line. The examination as to what he considers as an equivalent is to be conducted by the dean of the school. He can accept what he will, and he cannot be called to account for it. If he chooses to receive as an equivalent the ability to read and write, or even some practical knowledge in lieu of these, there is nothing to prevent it. So we are practically without any standard whatever, as far as the National Association is concerned. So long as it is in the power of deans to accept anything they may choose as an equivalent, any other regulations amount to nothing at all. There is not a representative of the objecting colleges who will not be ready to agree to any standard whatever, even though it be the diploma of a literary college, so long as he is given the power to say what he may accept as an equivalent.

Of course, this action cannot stand without disrupting the Association. The colleges which are determined to maintain a decent standard will never submit to have their diplomas placed upon the same level as those which practically have no standard. In the State of New York, for instance, the requirement for graduation demanded by law is the possession of a certificate of the successful completion of a full four years' course in, and graduation from, a High School or Academy, recognized as maintaining the standard of the New York schools. We know of one dental college which will not long affiliate with schools that do not maintain a decent standard. The University of Buffalo will not see its diplomas thus debased. It will take no precipitate action, for it proposes to continue the struggle for a better state of things. But if this should be found hopeless, there will in our opinion be no refuge for the schools and universities which propose to keep up their standard, save in the formation of an association among themselves, and the refusal to acknowledge the tickets and diplomas of the colleges that will not conform. They cannot afford to have their honors debased in the estimation of other professions, and in other countries.

But we do not believe that the majority of the colleges in our National Association seriously intend that such a state of affairs shall continue. We do not believe that any man worthy a place as an educator actually desires that opportunity shall be afforded for the admission into our ranks of really illiterate men. We have more faith in professional honesty than that would imply. There were influences at work at Old Point this year, which threw more than one ordinarily level-headed man completely off his balance. It was felt by many that the Association of Dental Examiners had taken unwarranted ground, and had assumed an attitude toward the colleges that was unauthorized. Conservative men had expected some kind of a compromise, and so long as that hope remained the tone of the college representative was toward an advance. It soon became manifest that the aggression was to continue, and that the conservative men were relying upon false expectations. There are few who will tamely submit to attempted coercion on the part of those not armed with proper authority. Threats and menacing actions only arouse opposition, and the natural result of the hostile attitude of the Board of Examiners was to bring about a reaction, and tend to defeat the very object which they say they have in view. They certaintly showed very little wisdom in arousing the deep feeling which was the consequence of their action, if their primal object is to raise the standard of dental education. There are men in the faculties who are as sincerely desirous of elevating the schools as is any man in the profession, but they will not be coerced by those to whom they owe no special allegiance. They will fight to the last gasp for their independence and liberty, even though in so doing they must take up arms against those with whom there should be amicable relations. We believe that very much of the reactionary movement of this year is due to the unwarranted and unwise interference of the Examiners Association, and that progress will not again be resumed until the Examiners become the coadjutors instead of the foes of the colleges.

There was another proposed advance that was defeated this year, and that was the proposition to increase the length of the term from six to seven months. That question was carried two years ago, but at the earnest solicitation of the representatives of two or three of the colleges the action was rescinded, upon the distinct understanding that this was solely to allow them time to make the change without disorganizing their schools. They asked but a year or two, and then they gave the assurance that they would no longer oppose the change. Yet they were quite as bitter in their opposition this year as ever. Now any reflective man must be convinced that the school that remains in session but half the time is not doing the work that it should. It was confidently expected that all the colleges which now have more than a six months term would vote for the extension. It was a distinct movement forward, it was in the line of progress, and it is incomprehensible that schools claiming a high standing should not heartily support the movement. The vote was taken by colleges, and it was lost by a bare majority. The records show the following to be the vote in favor of making the term seven months :—

| | |
|---|---|
| California University, - - | San Francisco, Cal. |
| Columbian University, - - | Washington, D. C. |
| Harvard University, - - | Washington, D. C. |
| National University, - - | Washington, D. C. |
| Chicago College Dental Surgery, - | Chicago, Ill. |
| Northwestern University - - - | Chicago, Ill. |
| Iowa University, - - - - | Iowa City, Iowa. |
| Boston Dental College, - - | Boston, Mass. |
| University of Michigan, - | Ann Arbor, Mich. |
| University of Buffalo, - - - | Buffalo, N Y. |
| New York College of Dentistry, | New York, N. Y. |
| Ohio College Dental Surgery, | Columbus, Ohio. |
| Western Reserve University, - | Cleveland, Ohio. |
| Pennsylvania Dental College, | Philadelphia, Pa. |
| University of Pennsylvania, - | Philadelphia, Pa. |

Against making the term seven months :—

| | |
|---|---|
| Birmingham Dental College, | Birmingham, Ala. |
| Atlanta Dental College, - - - | Atlanta, Ga. |
| Southern Medical College, - - - | Atlanta, Ga. |
| Louisville Dental College, - - | Louisville, Ky. |
| Baltimore College Dental Surgery, | Baltimore, Md. |
| Kansas City Dental College, - | Kansas City, Mo. |
| Western Dental College, - - | Kansas City, Mo. |
| Marion-Sims College, - - - | St. Louis, Mo. |
| Cincinnati College Dental Surgery, | Cincinnati, O. |
| Philadelphia Dental College, - | Philadelphia, Pa. |
| Tennessee Medical College, - | Knoxville, Tenn. |
| Central Tennessee College, - | Nashville, Tenn. |
| University of Tennessee, - - | Nashville, Tenn. |
| Vanderbilt University, - - | Nashville, Tenn. |
| University College, - - - | Richmond, Va. |
| Royal College of Ontario, - - | Toronto, Ont. |

Colleges absent and not represented :

| | |
|---|---|
| University of Denver, - - - | Denver, Col. |
| Northwestern Dental College, - | Chicago, Ill. |
| Indiana Dental College, - - | Indianapolis, Ind. |
| Detroit Medical College, - - | Detroit, Mich. |
| University of Medicine, - - | Cleveland, O. |

In the room but declining to vote :

| | |
|---|---|
| Harvard University, - - | Cambridge, Mass. |
| University of Minnesota, - | Minneapolis, Minn. |

The representative of Missouri Dental College, St. Louis, was not in the room.

The representative of the University of Maryland, Baltimore, was detained by sickness.

| | |
|---|---|
| Colleges voting aye, - - - | 15. |
| Colleges voting no, - - - | 16. |
| Present but not voting, - - | 2. |
| Representatives out of the room, - | 2. |
| Colleges unrepresented, - - | 5. |
| Total membership, - - - | 40. |

---

ARTICLE VIII.

